# GRUMB: a genome-resolved metagenomic framework for monitoring urban microbiomes and diagnosing pathogen risk

**DOI:** 10.1093/bioinformatics/btaf548

**Published:** 2025-09-26

**Authors:** Suleiman Aminu, AbdulAziz Ascandari, Rachid Benhida, Rachid Daoud

**Affiliations:** College of Chemical Sciences and Engineering (CCSE), Chemical and Biochemical Sciences (CBS), Mohammed VI Polytechnic University (UM6P), Benguerir, 43150, Morocco; Department of Biochemistry, Ahmadu Bello University, Zaria, Kaduna, 1096, Nigeria; College of Chemical Sciences and Engineering (CCSE), Chemical and Biochemical Sciences (CBS), Mohammed VI Polytechnic University (UM6P), Benguerir, 43150, Morocco; College of Chemical Sciences and Engineering (CCSE), Chemical and Biochemical Sciences (CBS), Mohammed VI Polytechnic University (UM6P), Benguerir, 43150, Morocco; Institut de Chimie de Nice (ICN), UMR CNRS 7272, Université Côte d’Azur, Nice, Provence-Alpes-Côte d’Azur, 06108, France; College of Chemical Sciences and Engineering (CCSE), Chemical and Biochemical Sciences (CBS), Mohammed VI Polytechnic University (UM6P), Benguerir, 43150, Morocco

## Abstract

**Summary:**

Urban infrastructure hosts dynamic microbial communities that complicate biosurveillance and AMR monitoring. Existing tools rarely combine genome-resolved reconstruction with ecological modeling and batch-aware analytics tailored to infrastructure-scale studies. We present GRUMB (Genome-Resolved Urban Microbiome Biosurveillance), an open-source, SLURM-compatible pipeline that reconstructs high-quality metagenome-assembled genomes (MAGs) from shotgun sequencing reads and integrates taxonomic/functional annotation (CARD, VFDB), batch-aware normalization, ecological diagnostics and machine learning classification of environment types with uncertainty and risk scoring. GRUMB accepts either SRA project accessions or paired-end FASTQ files with metadata, and produces assemblies, MAGs, taxonomic and functional profiles, ecological outputs and risk-informed classification. Its modular design enables reproducible, infrastructure-scale biosurveillance across diverse environments.

**Availability and implementation:**

GRUMB is freely available under the MIT License at: https://github.com/SuleimanAminu/genome-resolved-urban-microbiome-biosurveillance; Zenodo DOI: https://doi.org/10.5281/zenodo.15505402. Requirements: Linux (Ubuntu 20.04+), Python 3.11, R 4.2+, SLURM. Issues and feature requests are tracked on GitHub.

## 1 Introduction

The global urban microbiome is an emergent frontier in biosurveillance. With the expansion of cities and increased infrastructure density, built environments are increasingly acknowledged as dynamic sites for microbial exchange, pathogen spread, and AMR emergence (Forbes *et al.* 2017, Danko *et al.* 2021, Shen *et al.* 2022, Wu *et al.* 2023, Bruno *et al.* 2024). One Health-aligned priorities emphasizes the need for scalable, reproducible, genome-resolved platforms across different environments (New and Brito 2020, Salazar *et al.* 2022, Simon *et al.* 2023). However, from a bioinformatics standpoint, these datasets are challenging due to low biomass, contamination, heterogeneous sampling, and marked batch effects that degrade signal and hamper cross-environment comparisons (Wang and LêCao 2020, Shen *et al.* 2022, Aizpurua *et al.* 2024, Wang *et al.* 2025). These constraints motivate integrated workflows that explicitly model batch structure and deliver interpretable ecological and predictive outputs, i.e. genome-resolved methods that pair assembly and binning with ecological statistics and machine learning (Alneberg *et al.* 2020, Salazar *et al.* 2022, Vuong *et al.* 2022).

Despite rapid progress in metagenomics, current urban microbiome workflows remain limited in three respects (resolution, data complexity, and integration). In terms of resolution, reliance on 16S or shallow shotgun profiling yields coarse taxonomies that are insufficient for tracking pathogens and antimicrobial resistance (Pillay *et al.* 2022, Chklovski *et al.* 2023, Wu *et al.* 2023, Aminu *et al.* 2024). For data complexity, infrastructure samples are typically low biomass and contamination prone, with heterogeneous sampling and pronounced batch effects that, if not modeled, bias ecological estimates and downstream machine-learning results (Forbes *et al.* 2017, Pillay *et al.* 2022, Olsen and Riber 2025). For integration, even when metagenome-assembled genomes are reconstructed, many pipelines end at descriptive summaries rather than providing ecological diagnostics, uncertainty-quantified prediction, and risk-oriented outputs that support surveillance decisions (Sun *et al.* 2022, Bizzotto *et al.* 2024, Wijaya *et al.* 2024).

Several mature workflows address aspects of these limitations, particularly in assembly-based metagenomics. For instance, ATLAS provides a Snakemake pipeline that packages QC (BBTools), *de novo* assembly (metaSPAdes or MEGAHIT), depth-based binning (MetaBAT2 and MaxBin2 with optional DAS Tool), MAG QC (CheckM2), functional and taxonomic annotation (eggNOG, GTDB-Tk), plus MAG dereplication with dRep and per-sample abundance reports (Kieser *et al.* 2020). The nf-core/mag, on the other hand, offers a Nextflow workflow that standardizes read preprocessing (fastp, Bowtie2 with FastQC), assembly (MEGAHIT or SPAdes), binning (MetaBAT2), and summary QC (QUAST, BUSCO, and MultiQC), and taxonomy via GTDB-Tk or CAT/BAT (Krakau *et al.* 2022). In parallel, metaWRAP provides modular wrappers whose widely used strengths include “Bin refinement,” which consolidates outputs from multiple binners, and “Reassemble bins,” which recruits reads and reassembles each bin to improve contiguity (Uritskiy *et al.* 2018).

Despite their breadth, these frameworks are general-purpose rather than biosurveillance-specific. In particular, they typically do not integrate AMR and virulence screening linked to taxonomic context at contig and MAG levels, provide batch-aware species-matrix normalization for cross-site comparability, expose ecological diagnostics as first-class outputs (indicator species, NMDS, PERMANOVA, ANOSIM), or deliver uncertainty-aware machine learning with risk scoring tuned to high-contact urban settings.

**Figure 1.**
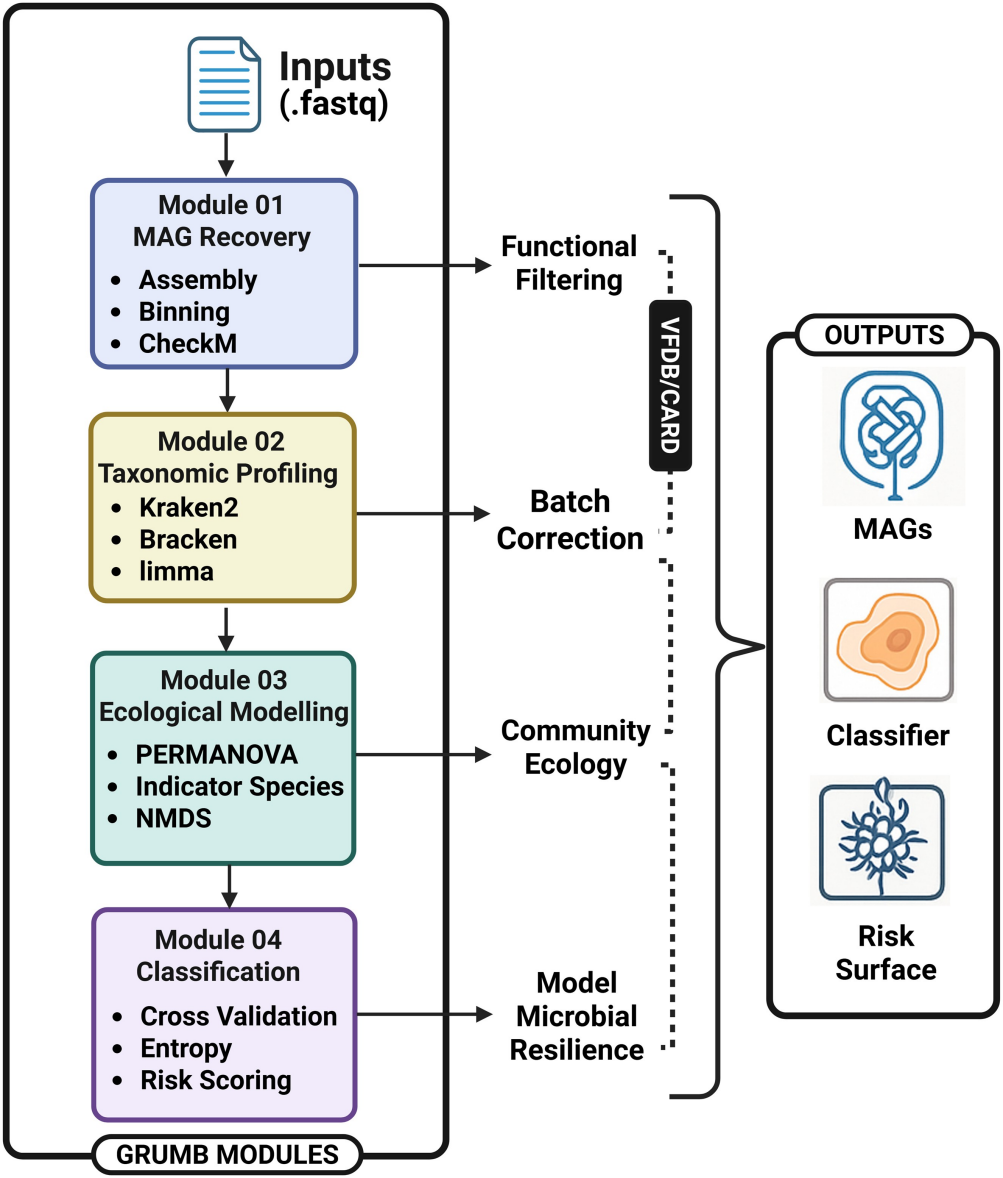
Overview of the GRUMB pipeline for genome-resolved urban microbiome biosurveillance. The pipeline comprises four modular stages: MAG recovery, taxonomic profiling with batch correction, ecological modeling, and machine learning-based classification. Functional filtering based on virulence and resistance genes (VFDB/CARD) informs ecological and predictive modeling, producing outputs including MAGs, classifiers, and microbial risk surfaces.

We present GRUMB (Genome-Resolved Urban Microbiome Biosurveillance), an open-source, SLURM-compatible pipeline purpose-built for infrastructure-scale surveillance (Fig. 1). The workflow reconstructs high-quality metagenome-assembled genomes from shotgun reads (metaSPAdes for assembly, MetaBAT2 for binning, CheckM2 for quality assessment), performs taxonomic and functional annotation (Kraken 2 and Bracken for species profiling; DIAMOND searches against CARD and VFDB), and builds analysis-ready species-abundance tables that include raw counts. The species-abundance tables are Hellinger transformed, and batch adjusted in order to stabilize cross-site comparisons. GRUMB computes ecological diagnostics such as diversity metrics, NMDS, PERMANOVA, ANOSIM, and indicator-species analyses, and trains supervised classifiers to predict environment types. The models report per-sample probabilities with entropy-based uncertainty and provide risk scores to support prioritization; an optional simulation module blends synthetic communities to probe contamination gradients and identity robustness. Inputs are SRA project accessions or paired-end FASTQ files with a metadata. The outputs include assemblies and MAGs with CheckM2 statistics, taxonomic profiles, functional hit tables, ecology and machine-learning outputs, as well as recorded configurations, software and database versions, random seeds, and logs to support reproducibility.

Together, these components integrate genome-resolved reconstruction with ecological statistics and predictive modeling in a single, reproducible workflow. The next section describes the architecture, inputs, and outputs in detail.

## 2 Implementation

GRUMB is a modular command-line workflow for genome-resolved metagenomics, built for infrastructure-scale biosurveillance. It integrates established tools from microbial genomics, ecological statistics, and machine learning into a reproducible, and scalable pipeline. The codebase uses Bash, Python, and R. Each module performs a well-defined task and can be extended, replaced, or parallelized. This architecture targets SLURM-managed clusters and also runs on workstations with sufficient resources. Conda environments are provided.

### 2.1 Modular architecture

1. *Module 01: Read preprocessing and MAG reconstruction (01_Bioinformatics/).* FastQC and BBMap Tools *(reformat.sh, bbmerge.sh, clumpify.sh, bbduk.sh)* for formatting, merging, duplicate removal, and adapter/quality trimming (Bushnell *et al.* 2017). FastQ Screen for contaminants screening(Wingett and Andrews 2018). MetaSPAdes assembly (Nurk *et al.* 2017); contig length filtering (seqtk); depth mapping; MetaBAT2 binning (Kang *et al.* 2019); CheckM2 quality control (Chklovski *et al.* 2023). This ensures that subsequent inferences rely on genome units that are both structurally coherent and biologically relevant.
2. *Module 02: Taxonomic, normalization, and AMR/virulence screening (02_Quality_batch_subsetting/).* Kraken 2 and Bracken species profiles (Lu *et al.* 2017, Wood *et al.* 2019); matrices exported as raw counts, Hellinger-transformed counts, and batch-adjusted matrices (limma::removeBatchEffect) when batch is available (Ritchie *et al.* 2015); DIAMOND searches against CARD and VFDB (default—evalue 1e-5,—max-target-seqs 1, sensitive mode) (Buchfink *et al.* 2015, Liu *et al.* 2019, Alcock *et al.* 2023); hits are merged into contig/MAG and species context to generate an AMR-focused subset. An optional minimum percent-identity threshold can be configured and applied during hit filtering.
3. *Module 03: Ecological analysis (03_Ecology/).* Alpha and beta diversity, NMDS ordination, PERMANOVA and ANOSIM (vegan) (Oksanen *et al.* 2001), and indicator-species analysis (indicspecies) for interpretable community structure across infrastructure types.
4. *Module 04: Predictive modeling and risk estimation (04_Machine_Learning/).* Model selection (using scikit-learn) by cross-validation, Monte Carlo evaluation and Feature importance (Pedregosa *et al.* 2011), Uncertainty and risk scoring where per-sample uncertainty is the normalized Shannon entropy of the predicted class-probability vector. A simple risk score equals (1 − entropy) × max (p) with an optional environment weight for prioritization. Scores are scaled to [0,1].

### 2.2 Data interfaces and outputs

GRUMB accepts either SRA project accessions retrieved via pysradb and the SRA Toolkit or paired-end FASTQ files, together with a minimal metadata table in TSV format. The pipeline produces assemblies and MAGs with CheckM2 completeness and contamination summaries, taxonomic profiles from Kraken 2 and Bracken, DIAMOND hit tables against CARD and VFDB at bin levels, analysis-ready species-abundance matrices in raw, Hellinger-transformed, and batch-adjusted forms, ecological reports for ordination and hypothesis tests, and machine-learning outputs including trained models, evaluation reports, per-sample probability matrices with entropy-based uncertainty, and risk scores. Tables 1 and 2 describe the detailed data interfaces and outputs obtained from GRUMB workflow.

**Table 1.** GRUMB Inputs and configuration.

Item	Required	Format/example	Purpose
Sequence data (paired-end)	Yes^a^	sample_R1.fastq.gz, sample_R2.fastq.gz	Primary input when not using SRA; QC, assembly, binning, profiling.
SRA project accession	Yes^a^	PRJNA123456	Alternative to FASTQ; fetched via pysradb and SRA Toolkit to generate FASTQ.
Metadata table	Yes	TSV with sample_id, environment_label, batch_id, location, collection_date, surface_type	Links reads to environments and batches; enables normalization and modeling.
Kraken2 database	Yes	Path to pre-computed DB	Read/MAG taxonomy with Kraken 2 and Bracken.
DIAMOND databases	Yes	Paths to CARD and VFDB DIAMOND DBs	AMR and virulence screening
Configuration	Recommended	YAML/JSON or shell variables	Paths and thresholds; threads; model settings.

a Provide either paired-end FASTQ **or** an SRA project accession.

**Table 2.** Key outputs produced by GRUMB.

Category	Files and formats	Produced by	Contents (examples)
Assemblies and MAGs	contigs.fasta, *.fa (bins)	metaSPAdes, MetaBAT2	Contigs and MAGs after length and quality filters.
MAG quality	checkm_quality_report.tsv	CheckM2	Completeness, contamination, lineage; passing MAG list.
Taxonomy	Kraken reports (*.report, *.screen), Bracken tables	Kraken 2, Bracken	Species names and abundances;
Function (ARG/virulence)	DIAMOND outfmt6 (*_CARD.csv, *_VFDB.csv) and merged TSVs	DIAMOND versus CARD/VFDB	Accessions, gene names, scores, e-values, percent identity; joined to contig/MAG and species.
Species matrices	counts_raw.tsv, counts_hellinger.tsv, counts_batch_adjusted.tsv	Normalization module	Sample × species tables for ecology and ML.
Ecology	ordination_coords.tsv, permanova.tsv, anosim.tsv, indicator_species.tsv; figures (.png/.pdf)	vegan, indicspecies	NMDS coordinates; test statistics and *P*-values; indicator taxa.
Machine learning	model.joblib, cv_results.tsv, evaluation.tsv, probabilities.tsv, entropy.tsv, risk_scores.tsv, feature_importance.tsv, figures (.png/.pdf)	ML module	Tuned model; grid search; per-repeat metrics; per-sample probabilities, uncertainty, risk; importance.
Reproducibility	config.json or YAML, software_versions.tsv, logs (.txt)	All modules	Parameters, database versions, random seeds, split assignments, run logs.

## 3 Evaluation

We validated GRUMB on publicly available short-read metagenomes from hospitals, ambulances, sewage systems, and public transport. The workflow reconstructed high-quality MAGs, produced species/functional matrices with batch correction, and generated ecological and predictive outputs. Machine-learning achieved consistent cross-validation performance, with entropy-based scores highlighting samples at higher ecological uncertainty. A companion manuscript reports detailed benchmarking while this Application Note focuses on tool implementation.

*Relationship to a companion study.* This Application Note documents the core GRUMB framework. A companion manuscript (currently under revision at Microbiome) applies GRUMB to a global metagenomic surveillance dataset and integrates several methodological extensions; including genome dereplication, species-resolved GTDB taxonomy, high-confidence ARG profiling using RGI, and virulence gene quantification via VFDB. To avoid redundancy, we present only a minimal demonstration here.

## 4 Conclusion

GRUMB unifies high-fidelity MAG reconstruction, batch-aware taxonomic harmonization, AMR/virulence screening, ecological diagnostics, and uncertainty-aware classification into a cohesive, reproducible workflow for infrastructure-scale biosurveillance. Inputs and outputs are explicit, with configurations, software/database versions, seeds, and logs recorded for full reproducibility. The modular, SLURM-compatible design makes GRUMB deployable on HPC or workstation settings across research and public-health domains. Recent extensions in a companion study demonstrate GRUMB’s flexibility for integrating custom pre-processing and profiling tools in biosurveillance contexts. By focusing on pathogen- and AMR-relevant features and producing interpretable ecological summaries, GRUMB provides a practical foundation for One Health surveillance of urban environments.

*Scope and limitations.* GRUMB targets short-read datasets but does not include long-read hybrid assembly or cross-project co-assembly by default. Genome dereplication, GTDB-based taxonomy, and curated ARG/VF profiling are supported in a modular companion implementation (see companion manuscript, under revision at Microbiome).

## Supplementary Material

btaf548_Supplementary_Data

## Data Availability

All datasets used in this study are publicly available. Accession numbers and project identifiers are provided in the Supplementary Material. The full pipeline and example data are accessible via GitHub and Zenodo.

## Software availability

GRUMB is open-source under the MIT License. Source code: https://github.com/SuleimanAminu/genome-resolved-urban-microbiome-biosurveillance. An archival snapshot of the exact version used in this manuscript is deposited at Zenodo (DOI: https://doi.org/10.5281/zenodo.15505402). System requirements: Linux (Ubuntu 20.04+), Python 3.11, R 4.2+, SLURM-compatible HPC; Documentation: See README.md.
